# Opioid Literacy in an Immigrant Group in Rural Area: Use of Social Determinant of Health Framework

**DOI:** 10.1093/geroni/igaa057.1277

**Published:** 2020-12-16

**Authors:** Hee Yun Lee, Eun Young Choi, Jieun Song, Jamie Gajos, Yan Luo

**Affiliations:** 1 University of Alabama, Tuscaloosa, Alabama, United States; 2 Leonard Davis School of Gerontology, Los Angeles, California, United States; 3 Waisman center, Madison, Wisconsin, United States; 4 The University of Alabama, Tuscaloosa, Alabama, United States

## Abstract

Opioid overdose risk is particularly high in immigrant communities partly due to limited English proficiency (Guarino et al., 2015). Previous studies reported that social determinants of health (SDH) have been associated with risk for opioid overdose (Dasgupta et al., 2018). The current study examines the association between SDH and literacy of opioid overdose risk among the immigrant population living in a rural area. Specifically, we examine the association in various age groups including young adults (aged 20 to 34), middle-aged (aged 35 to 49), and older adults (ages 50 to 75). Data were drawn from a sample of Korean American immigrants residing in rural Alabama (N=225). The participants administered the Brief Opioid Knowledge (BOOK) Questionnaire (Dunn et al., 2016). Multiple regression analyses were conducted for three age groups to identify predictors of opioid literacy. Overall, older adults had lower levels of opioid literacy relative to their younger counterparts. Among young adults, low English proficiency, more chronic conditions, and greater depressive symptoms were significant predictors of limited opioid literacy. For the middle-aged adults, lower levels of health literacy and more pain symptoms were associated with limited opioid literacy. Among older adults, women, those with higher English proficiency, and lower health literacy had lower levels of opioid literacy. The findings demonstrated a greater vulnerability of older immigrants to limited opioid literacy. Different predictors based on SDH of limited opioid literacy across age groups have implications for tailored health promotion strategies to reduce opioid overdose risk.

